# 2-Thiouridine formation in *Escherichia coli*: a critical review

**DOI:** 10.1128/jb.00420-24

**Published:** 2024-12-11

**Authors:** Silke Leimkühler

**Affiliations:** 1 Department of Molecular Enzymology, Institute of Biochemistry and Biology, University of Potsdam 163276 , Potsdam, Brandenburg, Germany; University of Virginia School of Medicine, Charlottesville, Virginia, USA

**Keywords:** sulfur, tRNA modification, thiouridylase, Fe–S cluster

## Abstract

Modifications of transfer RNA (tRNA) have been shown to play critical roles in the biogenesis, metabolism, structural stability, and function of RNA molecules, and the specific modifications of nucleobases with sulfur atoms in tRNA are present in prokaryotes and eukaryotes. The s^2^ group of s^2^U34 stabilizes anticodon structure, confers ribosome-binding ability to tRNA, and improves reading frame maintenance. In particular, specific enzymes catalyze the biosynthesis of sulfur-containing nucleosides of s^2^U34, such as the L-cysteine desulfurase IscS and the tRNA thiouridylase MnmA in *Escherichia coli*. Until recently, the mechanism of sulfur transfer in *E. coli* was considered to involve persulfide chemistry; however, a newly proposed mechanism suggests the involvement of a [4Fe–4S] cluster bound to MnmA. This review provides a critical appraisal of recent evidence for [4Fe–4S]-dependent or [4Fe–4S]-independent tRNA thiolation in 2-thiouridine formation.

## INTRODUCTION

Precise decoding of the genetic code at the ribosome is a fundamental process for all living organisms, which minimizes the alteration of the translated protein. Nucleoside modifications are introduced posttranscriptionally into the RNAs of all organisms. Transfer RNAs (tRNAs) play a critical role in protein synthesis by translating codons on messenger RNAs (mRNAs) into the corresponding amino acids in the ribosome. All cellular RNAs feature posttranscriptional chemical modifications, which are evolutionarily well-conserved (1). Sulfur modifications on tRNA occur in many cellular processes (2–4). They stabilize the tertiary structure and introduce recognition determinants for the ribosome, resulting in a more accurate decoding process and minimizing frame-shifting (5, 6). Sulfur modification of nucleosides is also common but has been observed mostly in tRNAs. The uridine at wobble position 34 in tRNA^Lys^, tRNA^Glu^, and tRNA^Gln^ from bacteria and eukaryotes contains sulfur at the 2-position (s^2^U34), which is further modified to 5-methylaminomethyl-2-thiouridine (mnm^5^s^2^U) or 5-carboxymethyaminomethyl-2-thiouridine (mcm^5^s^2^U) in *Escherichia coli* (2, 7, 8). Structurally, the sulfur at position 2 and hypermodification at position 5 act to stabilize the C3-endo conformation of the ribose ring (6, 9). This increases conformational rigidity and minimizes wobble base-pairing with guanosine at the third position of the codons *in vitro*. Mutants lacking s^2^U display a significant increase in frame-shifting, a consequence common to many tRNA modification mutants (10). Thiolation at position 2 is also essential for the tRNAs for Lys, Gln and Glu, allowing the modified mnm^5^s^2^UUU to interact with AAA- or AAG in the A-site of ribosomes (11–13).

## IDENTIFICATION OF THE MNM^5^S^2^U TRNA THIO-MODIFICATION IN *E. COLI*

MnmA is the thiouridylase that catalyzes 2-thiolation of uridine at position 34 in *E. coli* (14). A reverse genetic approach combined with mass spectrometry was used by the Suzuki group to identify the complex sulfur-transfer system for 2-thiouridine formation on tRNAs in *E. coli* (8, 15, 16). For the formation of (c)mnm^5^ s^2^ U on the tRNAs for Lys, Gln, and Glu in *E. coli*, a sulfur-relay system was identified, which includes the initial sulfur mobilization by the L-cysteine desulfurase IscS and the proteins TusA, TusBCD, TusE, and MnmA (8). The proteins TusA, the TusBCD complex, and TusE are used to transfer sulfur from the IscS-bound persulfide and generate a persulfide on TusE, which serves as the sulfur source for s^2^U34 thiolation of the tRNA bound to MnmA (17). *In vitro*, the presence of the Tus proteins increases the tRNA thiolation activity 200-fold in comparison to that of MnmA alone (8, 14). However, MnmA and IscS are sufficient to transfer the sulfur for s^2^U formation *in vitro. In vivo,* MnmA is essential for this step, but it is not essential for the viability of *E. coli* (14).

**Fig 1:**
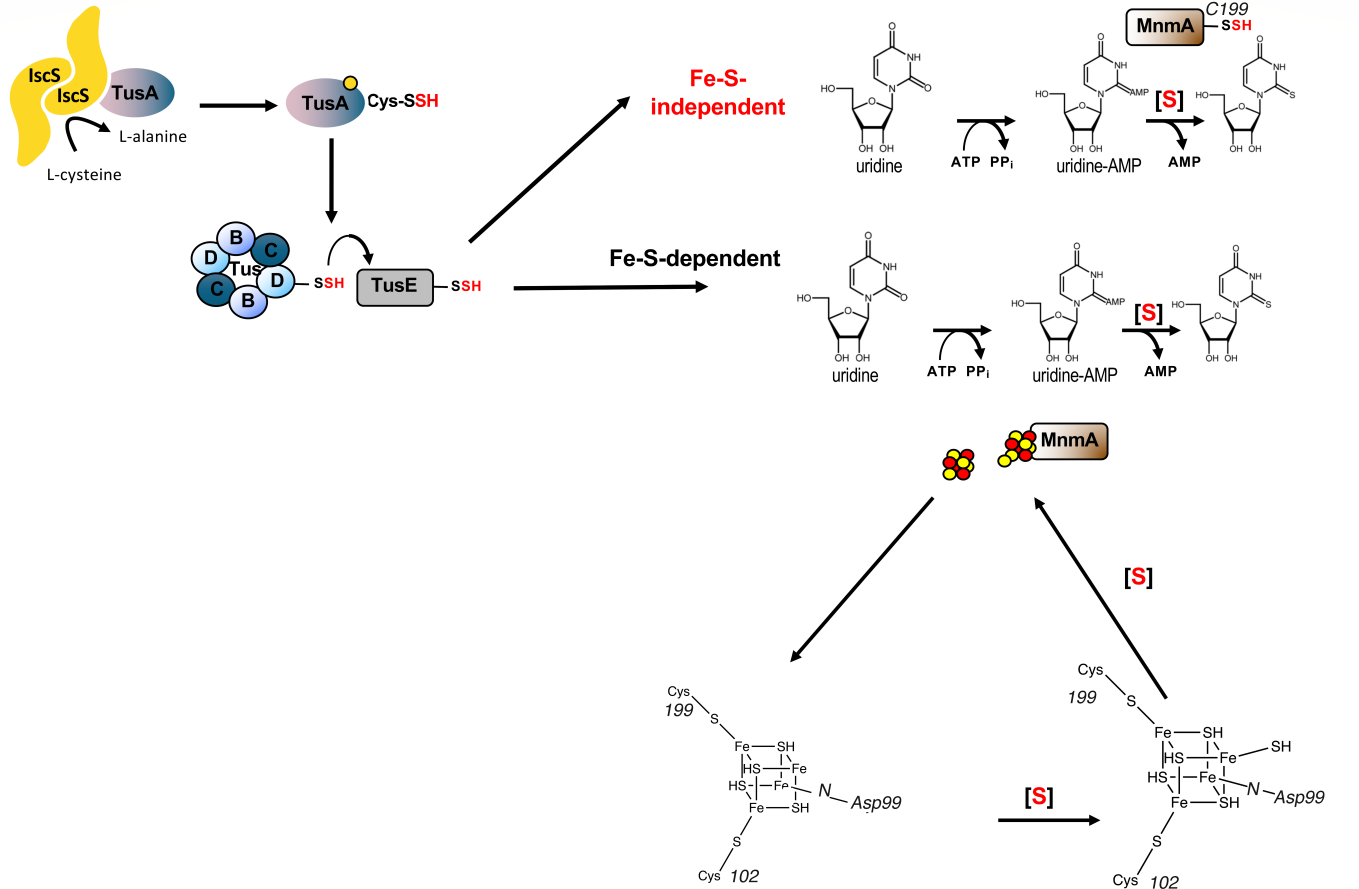
Sulfur is transferred from IscS to TusA and then in a sulfur relay via TusD, TusE, and MnmA to the tRNA U34 that has been activated by MnmA under ATP consumption. The sulfur is then transferred from the MnmA persulfide to U34 in the tRNA to form s^2^U34. In an alternative mechanism, the sulfur from TusE is transferred to the [4Fe–4S] cluster on MnmA, forming an [4Fe–5S] cluster. The sulfur from this cluster is then transferred to the activated U34-AMP in tRNA, forming s^2^U34.

Using a reverse genetic approach, TusA, the TusBCD complex, and TusE, in addition to MnmA, were identified to be involved in s^2^U formation (8). First, TusA interacts with the L-cysteine desulfurase IscS to accept the persulfide, which directs the sulfur flow to this pathway (Fig. 1) (18, 19). MnmA then accepts the persulfide sulfur on a conserved Cys199 residue in its active site after its transfer from TusA via TusD and TusE (Fig. 1) (8). However, in some species, such as in *Bacillus subtilis*, there is no need for such intermediate persulfide carrier proteins (20).

Overall, MnmA possesses a PP-loop motif and is a member of the ATP-pyrophosphatase family (15). This enzyme utilizes a two-step mechanism to form an adenylated tRNA intermediate (15, 21), whereby it has been suggested that a nucleophilic attack by the persulfide sulfur on MnmA generates tRNA s^2^U34 and releases AMP (21). The modification enzyme ThiI, which is involved in s^4^U synthesis, also contains a PP-loop and utilizes a similar two-step mechanism (22–24). A snapshot of s^2^U formation via the acyl-adenylated intermediate has been revealed in the co-structure of the *E. coli* MnmA–tRNA complex (21). Based on this structure, and in analogy with the mechanism of ThiI, it was proposed that the uridine 34 of the bound tRNA reacts with ATP to form an acyl-adenylated intermediate. This then reacts with the terminal sulfur released from the persulfide of MnmA-Cys199 with assistance from the conserved Cys102 residue (21, 25). The adenylated intermediate has been trapped and visualized in the crystal structure of *E. coli* MnmA in complex with tRNA (15, 21). Notably, however, the cysteine desulfurase YrvO of *Bacillus subtilis* is able to transfer the L-cysteine sulfur directly to MnmA but only in the presence of ATP (20).

Adopting a reverse genetic approach, it was revealed that s^2^U formation in *E. coli* involves a more complex cascade of sulfur-carrier proteins consisting of TusA, the TusBCD complex, and TusE, in addition to MnmA and IscS (26). It has been proposed that, in the final step on a sulfur-transfer pathway, a persulfide on the catalytic cysteine of TusE is transferred to the catalytic Cys199 of MnmA, forming a persulfide, which then acts as the sulfur that is transferred to the activated tRNA on MnmA (Fig. 1). Nevertheless, in an *in vitro* experiment using labeled [^35^S]-L-cysteine together with all of the Tus proteins, *E. coli* MnmA remained barely labeled (26). Consequently, proof that TusE transfers the persulfide directly to MnmA in *E. coli* remains to be definitively provided.

The mechanism proposed for *E. coli* MnmA (26) involves three conserved residues (Asp99, Cys102, and Cys199) (Fig. 1), which target U34 of the bound tRNA; is based on the structural analysis of *E. coli* MnmA in complex with tRNA (17); and has analogy to the proposed mechanism for *E. coli* ThiI. Studies with cysteine variants C102S and C199A, as well as a D99A variant, of MnmA showed that these proteins were unable to transfer the sulfur from L-cysteine to the tRNA (8). The formation of a persulfide on Cys199 would enable Cys199-SSH to act as a nucleophile to attack the C2 position of the adenylated tRNA intermediate bound to MnmA, followed by the cleavage of the disulfide bond between the enzyme and the tRNA by Cys102, assisted by Asp99 acting to deprotonate/protonate the N3 atom of U34 (21, 26). However, this proposed persulfide-based mechanism still needs to be demonstrated for the MnmA of *E. coli*.

## FORMATION OF THE CMNM^5^ AND MNM^5^ SIDECHAIN MODIFICATIONS

The 5-methyluridine (xm^5^U34)-derived modifications are additionally required for the accurate translation of codons ending in A or G in the mixed-codon family boxes. The first step in the biosynthesis of xm^5^U34 modifications in bacteria like *E. coli* is the formation of a 5-carboxymethylaminomethyl (cmnm^5^) group on C5 of the uridine by the enzymes MnmE and MnmG. The MnmEG complex catalyzes two different GTP- and FAD-dependent reactions, which produce either 5-aminomethyluridine (nm^5^U) or 5-carboxymethylaminomethyluridine (cmnm^5^U34) by using ammonium or glycine, respectively, as substrates. The cmnm^5^ group may be further converted to 5-methylaminomethyl (mnm^5^) by the bifunctional enzyme MnmC in bacteria. In this reaction, MnmC catalyzes the cleavage of the carboxymethyl group of cmnm to generate 5-aminomethyl (nm^5^) (27). Alternatively, MnmEG is also suggested to install nm^5^U *in vitro* and *in vivo*. MnmE is a GTPase that binds to multiple tetrahydrofolate (THF) derivatives (28–30), while MnmG is an oxidoreductase that binds FAD and NADH (31, 32). *In vitro*, MnmE and MnmG are proposed to form an α_4_β_2_ complex that utilizes FAD, NADH, and K^+^ as cofactors; N_5_-N_10_-methylenetetrahydrofolate (CH_2_ THF), glycine (Gly), and tRNA as the substrates; and together they catalyze the guanosine-5′-triphosphate (GTP) hydrolysis-dependent modification of tRNA (32). In the proposed mechanism, FADH_2_ forms a covalent adduct with the methylene group on CH_2_-THF to form an iminium intermediate, which is then transferred to the uridine base and undergoes addition with the nucleophilic substrate (glycine) to the cmnm moiety (27, 29, 33). A Cys residue from the proteins has been proposed to play a key role in activating the uridine for methylene transfer. A glycine moiety is then proposed to be attached to the methylene U in MnmEG (34). The precise nature of the biochemical steps driven by GTP binding and hydrolysis remains unknown. Isolation of mutants containing mnm^5^ or s^2^ modifications at U34 suggested that these modifications occur independently of each other (31, 35, 36); thus, thiolation may either precede or follow the synthesis of the side-chains at position 5. So far, the possibility that the presence of the s^2^ group may facilitate the modification of position 5 by modulating the electron density distribution in the uridine ring has not been explored.

## 2-THIOURIDINE FORMATION: AN FE–S CLUSTER-DEPENDENT OR -INDEPENDENT PATHWAY?

Until recently, it has been consistently believed that s^2^U formation in *E. coli* occurs in an Fe–S cluster-independent manner. Suzuki initially reported a twofold reduction in s^2^C synthesis and an approximate tenfold reduction in the levels of the ms^2^io^6^A modification in *Salmonella typhimurium* strains carrying mutations in the *iscU*, *hscA*, or *fdx* genes (involved in Fe–S cluster formation by the ISC pathway (37), while the levels of s^4^U or (c)mnm^5^s^2^U modifications remained unaffected by the mutations. This then led Suzuki to suggest that two principally distinct routes for thiolation of tRNA exist: the [Fe–S]-independent route for s^4^U and (c)mnm^5^s^2^U formation and the [Fe–S]-dependent route for the synthesis of s^2^C and ms^2^io^6^A (16). Later, the sulfur mobilization system, SUF, was reported as a second [Fe–S] cluster biosynthetic machinery under oxidative stress conditions and under iron-limitation conditions (18). It was subsequently shown that the mnm^5^s^2^U or s^4^U tRNA modifications are independent of the presence of the SUF system in *E. coli,* while the ms^2^i^6^A37 and s^2^C32 modifications were recovered when the *suf* operon was overexpressed in a Δ*iscU* or a Δ*iscS* mutant (18). This finding supported the hypothesis that the biosynthetic pathways for the s^2^U and s^4^U tRNA modifications are [Fe–S] cluster-independent in *E. coli*. This also showed that the SUF system could indeed provide the [Fe–S] clusters necessary for the ms^2^i^6^A37 and s^2^C32 thio-modifications (18). Therefore, the tRNA thio-modifications of 2-thiocytidine at position 32 (s^2^C32) and of 2-methylthio-N6-isopentenyladenosine at position 37 (ms^2^i^6^A37) for certain tRNAs are [Fe–S] cluster-dependent (16). In *E. coli*, these tRNA modifications require the proteins TtcA for the s^2^C32 modification and MiaB for the ms^2^i^6^A37 modification. TtcA binds one [4Fe–4S] cluster, while MiaB binds two [4Fe–4S] clusters and is a member of the radical-SAM superfamily of proteins (38, 39). So far, MnmA homologs have been divided into two groups by Shigi (40), and these are defined by the presence of a C-DxxC-C or CC-CxxC-C motif, respectively, in the protein. Consequently, MnmA proteins are divided into D- and C-type classes. Although D-type MnmAs are most commonly found in Proteobacteria (e.g., in *Escherichia* and *Pseudomonas* species), a C-type MnmA variant lacking the region containing the first CC motif is found in *Campylobacter* and *Helicobacter* (40).

In addition, *E. coli* MnmA has been considered to be an [Fe–S] cluster-independent protein based on the fact that no [Fe–S] cluster was identified in the crystal structure of the protein (15). However, recently, a report describing the purification of a [4Fe–4S] cluster-containing MnmA protein isolated under anaerobic conditions from *E. coli* has led to the proposal that this [4Fe–4S] cluster is essential for its activity (41), which has also been reviewed in (42). This report contradicts the previously published data, which showed that in an *E. coli* Δ*iscS* mutant strain carrying an *suf*-expressing plasmid, the strain regained the ability to produce s^2^C32 or ms^2^i^6^A37-modified tRNAs, but not the s^4^U8 and (c)mnm^5^s^2^U34 tRNA modifications (18).

This new discovery led Zhou et al. to investigate whether *E. coli* MnmA could also be a [4Fe–4S]-dependent enzyme (41). When MnmA was over-produced and purified anaerobically (41), it was noted after nickel affinity chromatography that MnmA had a brownish color and showed a weak absorption at 410 nm that bleached under aerobic conditions, suggesting the presence of an O_2_-sensitive [4Fe–4S] cluster. They then confirmed that MnmA was able to bind a [4Fe–4S] cluster by UV–visible and EPR spectroscopy after anaerobic cluster reconstitution. While the aerobically purified protein had the tendency to form a dimer, anaerobically purified [4Fe–4S] cluster-containing holo-MnmA was a monomer. Thus, the oligomeric states of apo- and holo-MnmA indicated that the cluster does not require dimerization. The thiolation activity of MnmA was also tested by quantifying s^2^U formation after separation by HPLC. Their assay used an *E. coli* tRNA^GluUUU^ transcript together with inorganic sulfide as the sulfur source, and after subsequent hydrolysis, the nucleosides could be quantified. The activity of the [4Fe–4S] cluster-containing protein was measurably higher in this assay. In contrast to the wild-type MnmA enzyme, mutant *mnmA* genes encoding D99A-MnmA, C102A-MnmA, and C199A-MnmA variants showed that the corresponding strains were unable to restore U34 thiolation in *in vivo* complementation experiments of a Δ*mnmA* strain (41). Based on these findings, the group proposed that Asp99, Cys102, and Cys199 are the ligands of the [4Fe–4S] cluster (Fig. 1). Using an Alphafold model of *Thermus thermophilus* MnmA, which was superimposed onto a structural model of the crystal structure of *E. coli* MnmA in complex with adenylated tRNA, they proposed that the three cysteines that bind the [4Fe–4S] cluster in *Thermus thermophilus* MnmA are positioned very closely to the three residues proposed to be the catalytic residues in *E. coli* MnmA (42). Accordingly, a new [4Fe–4S]-dependent mechanism was proposed by Zhou et al. (41) for *E. coli* MnmA (Fig. 1) (42). In this model, the persulfide–sulfur from TusE-SSH would be transferred to the [4Fe–4S] cluster of MnmA, forming an [4Fe–5S] cluster (Fig. 1) (42). However, the mechanism of release of the sulfur atom from the persulfide is unclear in their model, in addition to the highly hypothetical [4Fe–5S] cluster on MnmA and the subsequent transfer of this sulfur to tRNA. The authors gave several reasons why the [4Fe–5S] cluster had been missed in previous studies of *E. coli* MnmA. First, the protein had been purified aerobically; thus, the cluster was degraded and lost. Second, the use of a strain overexpressing *E. coli* MnmA may have led to MnmA with an incomplete content of the [4Fe–4S] cluster. The authors also suggest that the [4Fe–4S] cluster bound by two cysteines and an aspartate is likely much less stable than those coordinated by four cysteines. The authors therefore concluded that in previous studies, MnmA with an incomplete loading of the [4Fe–4S] cluster resulted in its reported low catalytic activity (14, 26). These are valid arguments as to why, in the past, a [4Fe–4S] cluster might have been missed on this protein. However, applying an Alphafold model structure to assume [4Fe–4S] cluster-binding constitutes a bias toward homologs in which [4Fe–4S] clusters have been structurally confirmed. Therefore, although highly plausible, without experimental (biochemical or structural) confirmation of the presence of the [4Fe–4S] cluster in MnmA, these data should be treated with caution. Furthermore, a more recent report by Olalekan et al. (43) contradicts the findings by Zhou et al. (41) and shows that their [4Fe–4S]-reconstituted MnmA protein is inactive and does not transfer sulfur to tRNAs, as shown in an experiment using [^35^S]-labeled L-cysteine. Further, it was shown that the [Fe–S]-containing protein was still able to bind tRNA or ATP, but the interaction with IscS was impaired by the presence of the cluster, thereby negatively influencing sulfur transfer to MnmA. Finally, in a similar experiment to that used by Zhou et al. (41), different results were obtained by Ogunkola et al. (43). By using a mutant strain that does not produce [Fe–S] clusters (a Δ*iscUA*/Δ*suf* strain), Zhou et al. reported that s^2^U formation was similar in this strain as compared to the wild-type strain (41). However, this conclusion was not quite correct, as the cmnm^5^S^2^U levels were only 64% of those measured for the wild-type strain, while the mnm^5^s^2^U levels were increased by 25% compared to those of the wild-type strain. Actually, these levels were apparently comparable to the ones obtained for a Δ*ttcA* strain shown in their report, but not for the wild-type. In contrast, Ogunkola et al. complemented the [Fe–S] cluster assembly-defect in the *isc-suf* double null mutant by introduction of the *suf*-operon on a plasmid, which rescued s^2^C and ms^2^i^6^A37 synthesis (as both are [Fe–S] cluster-dependent), but neither s^2^U nor s^4^U synthesis, as synthesis of both is proposed to be [Fe–S] cluster-independent) (43). Zhou et al. interpreted their results by proposing that an unknown [Fe–S] cluster biogenesis system exists, which inserts the [Fe–S] cluster into MnmA for s^2^U synthesis in this strain (41). However, it is conceivable that the researchers accumulated a suppressor mutation in their *isc-suf* double null mutant because the strain grows very poorly and only in the presence of mevalonate. Moreover, since the Δ*ttcA* mutant strain showed the same cmnm^5^s^2^U and mnm^5^s^2^U levels as the [Fe–S] cluster-deficient strain, the lack of a s^2^C modification seems to influence the s^2^U modification. The criticism of Gervason et al. (42) was that in the Ogungola report (43), the *in vitr*o formation of the S^2^U-modified nucleosides is very small. However, a difference was seen between no peaks with the Fe–S reconstituted enzyme and small peaks with the Fe–S free enzyme. These apparent differences in experimental findings need to be investigated and clarified in future studies.

Another question that arose based on the experimental findings reported by Zhou et al. (41), was that their “apo-MnmA” also yielded a low level of enzyme-dependent tRNA-thiolation product (see Fig. 2C, lanes 2 and 3 in reference [41]). Why should apo-MnmA show any enzyme activity, if MnmA apparently requires an [Fe–S] cluster? This rather contradictory observation highlights a further issue that needs to resolved in future studies.

## HOW TO SOLVE THE PROBLEM WITH THE CONTRADICTORY RESULTS?

Meanwhile, it is clear that a [4Fe–4S] cluster can be reconstituted *in vitro* into MnmA, as has been confirmed by multiple research groups (41, 43). However, it should also be considered that a [2Fe–2S] cluster is bound to some extent by MnmA and that this might contribute to, or inhibit, the activity of the protein. One group has claimed that a [4Fe–4S] cluster is required for activity, while another group has shown that the cluster-bound protein is inactive and only the cluster-free protein shows activity. Both research groups used MnmA that had its [Fe–S] cluster reconstituted *in vitro*. Moving forward, it would be mandatory to show that the protein also binds a [4Fe–4S] cluster *in vivo* and that this contributes to its activity. Zhou et al. (41) have so far not considered that the binding of the [4Fe–4S] cluster to MnmA might be a way to regulate the activity of the protein in dependence on the oxygen availability, e.g., like an “oxygen switch.” In the cell, [Fe–S] clusters will not be inserted into MnmA when oxygen is present since they are not typically produced under these conditions, and this lack of cluster would then lead to an active MnmA protein. The current data state that under anaerobic conditions, [Fe–S] clusters are produced and inserted into MnmA, which results either in active MnmA (Zhou et al. [41]) or in inactive MnmA (Ogunkolan et al. (43). Further, oxygen as ROS also would remove the sulfur from the thionucleosides of tRNA, and with MnmA becoming active under these conditions by the loss of the Fe–S cluster, MnmA might even be involved in the repair of dethiolated tRNAs. This might be a way to ensure accurate translation under oxidative stress conditions. However, it has not been considered that this might also be a way to direct [Fe–S] cluster insertion such that a particular pathway for tRNA thiolation is activated. For example, if MnmA remains inactive, the other [Fe–S] cluster-dependent tRNA thiolation enzymes might become more important, like TtcA or MiaB, so that [Fe–S] cluster insertion, and thus sulfur for tRNA modification, is directed to these pathways. This might also provide an explanation for the observation that a Δ*ttcA* mutation also seems to influence s^2^U formation in *E. coli*. This proposal should be considered in future studies.

## OPEN QUESTIONS ABOUT THE MECHANISM OF S^2^U FORMATION

So far, the mechanism of s^2^U formation has not been resolved. Two different mechanisms have been proposed (Fig. 1), one being based on persulfide chemistry and the other one proposing the formation of a [4Fe–5S] cluster (42); however, no direct experimental evidence for either of these mechanisms exists. The tRNA thiolation enzymes requiring an [Fe–S] cluster for catalysis are a recent discovery (40, 44), and the functional relevance of this cluster might have been missed in previous studies (14, 15, 21). Because the cluster is labile under aerobic conditions, this would result in a lack of, or only partial, insertion into MnmA during aerobic reconstitution experiments and would explain why some tRNA thiolation still apparently occurs *in vitro*. The functional requirement of the [Fe–S] cluster for productive tRNA thiolation needs to be demonstrated *in vivo*, and not only *in vitro*, since several proteins are known to bind [Fe–S] clusters nonspecifically following chemical reconstitution. Furthermore, data accrued using site-directed mutagenesis studies exchanging individual cysteines ligating the cluster may not lead to definitive conclusions, as an [Fe–S] cluster can also be bound by only three cysteines, or other amino acids can substitute for them to facilitate cluster ligation. A persulfide can also be generated nonspecifically on a cysteine, or the sulfur can be acquired from free sulfide in solution. Thus, *in vivo* studies that could definitively show the presence and involvement of an [Fe–S] cluster or a persulfide on MnmA, likely by the aid of radioactively labeled sulfur, would provide the most conclusive evidence to prove which of the proposed mechanisms is correct. The analysis of mutant strains often can be used to help clarify a hypothesis, but often not only one pathway is influenced in a mutant strain, but several pathways can also be altered, and therefore the results should be treated with caution. Studies pertaining to the native strain are suggested, combined with proteomics or native mass spectrometry, to reveal the nature of the cluster or persulfide bound to MnmA under conditions that allow s^2^U formation. A crystal structure of the natively and anaerobically purified enzyme containing an [4Fe–4S] cluster would help in providing the definitive resolution of mechanism.
